# Intensity-modulated radiotherapy with carbon ion boost for high-risk sinonasal squamous cell carcinoma: clinical outcomes and the management of the node-negative neck

**DOI:** 10.1016/j.ctro.2025.101064

**Published:** 2025-10-27

**Authors:** Lukas Bauer, Katharina Weusthof, Sebastian Regnery, Maximilian Deng, Philipp Schröter, Florian Stritzke, Nils Netzer, Henrik Franke, Kristin Uzun-Lang, Julius Moratin, Oliver Ristow, Rubens Thoelken, Semi B. Harrabi, Klaus Herfarth, Sebastian Adeberg, Jürgen Debus, Thomas Held

**Affiliations:** a Heidelberg University, Medical Faculty Heidelberg, Department of Radiation Oncology, Heidelberg University Hospital, Heidelberg, Germany; b Heidelberg University, Medical Faculty Heidelberg, Department of Radiation Oncology, Heidelberg University Hospital, Heidelberg Institute of Radiation Oncology (HIRO), Heidelberg, Germany; c National Center for Tumor Diseases (NCT), Heidelberg, Germany; d Clinical Cooperation Unit Radiation Oncology, German Cancer Research Center (DKFZ), Heidelberg, Germany; e Heidelberg Ion Beam Therapy Center (HIT), Heidelberg, Germany; f German Cancer Consortium (DKTK), Partner Site Heidelberg, German Cancer Research Center (DKFZ), Heidelberg, Germany; g Heidelberg University, Medical Faculty Heidelberg. Department of Oral and Maxillofacial Surgery, University Hospital Heidelberg, Heidelberg, Germany; h Department of Radiotherapy and Radiation Oncology, Marburg University Hospital, Marburg, Germany; i Marburg Ion-Beam Therapy Center (MIT), Department of Radiotherapy and Radiation Oncology, Marburg University Hospital, Marburg, Germany; j Universitäres Centrum für Tumorerkrankungen (UCT) Frankfurt - Marburg Germany, Marburg, Germany; k Heidelberg University, Medical Faculty Heidelberg, Department of Otorhinolaryngology, University Hospital Heidelberg, Heidelberg, Germany

**Keywords:** Head and neck cancer, Paranasal sinuses, Squamous cell carcinoma, Carbon ion radiotherapy, Toxicity, Local control, Neck dissection, Lymph node metastases

## Abstract

**Background:**

Local control of high-risk sinonasal squamous cell carcinoma (SCC) remains challenging and often involves significant morbidity. Management of the node-negative neck is still debated. This study evaluated treatment outcomes, neck management strategies, and toxicity rates in patients treated with intensity-modulated radiotherapy (IMRT) combined with a carbon ion boost.

**Methods:**

We evaluated 43 patients with sinonasal SCC treated with IMRT and carbon ion boost between 2011 and 2019. Follow-up followed head and neck cancer guidelines. Patient and tumor characteristics, treatment outcomes, and toxicity (per CTCAE v5.0) were assessed. Statistical analysis was performed using R version 4.1.0.

**Results:**

Median age was 64 years (range 19–83). Most patients had T3–4 tumors (n = 36; 83.7 %) and were clinically node-negative (n = 40; 93.0 %). The predominant regimen was 24 Gy (RBE) carbon ion boost in 8 fractions, followed by 50 Gy IMRT in 25 fractions. Postoperative RT was delivered to 25 patients (58.1 %), while 18 (41.9 %) received definitive RT. Elective or therapeutic neck dissection was performed in 10 patients (23.3 %), none showing lymph node metastases. In total, 22 distinct cervical lymph node treatment strategies were applied. Median follow-up for overall survival (OS) was 25.1 months. Tumor recurrence occurred in 11 patients (25.6 %), primarily due to local failure (n = 7; 63.6 %). OS and local progression-free survival (L-PFS) at 12/24 months were 93.0 %/89.3 % and 88.4 %/79.0 %, respectively. Grade III acute and late toxicity occurred in 9.3 % and 4.7 % of patients, respectively. No grade IV toxicity was reported. Mean numbers of grade I–II toxicities per patient were 4.8 (acute) and 2.4 (late).

**Conclusion:**

IMRT with carbon ion boost provides promising OS and local control in high-risk sinonasal SCC. The management of the node-negative-neck was highly individualized. Toxicity was acceptable and may be further reduced by tailoring cervical lymph node treatment in selected patients.

## Highlights

• Carbon ion boost with IMRT shows high local control in sinonasal SCC.
• Node-negative neck management remains variable in sinonasal SCC treatment.
• Treatment shows low grade III toxicity with no grade IV events observed.
• First clinical outcomes of combined IMRT and carbon ions for sinonasal SCC.
• Study supports individualized neck treatment to reduce radiation toxicity.

## Introduction

Sinonasal cancer, comprised of a heterogenous group of different tumor entities, constitutes around 3 % of all head and neck cancers [1]. The most common histology (50–60 %) is squamous cell carcinoma (SCC) [2], which may show overlapping morphological and functional features with other histological subtypes, e.g. sinonasal undifferentiated carcinoma [3]. Due to the rarity of early symptoms, sinonasal cancer is often diagnosed at an advanced tumor stage, requiring a multidisciplinary treatment approach [4]. In general, local recurrence after surgery and postoperative radiotherapy (RT) occurs within five years post-treatment in around half of the patients [5]. However, the therapeutic options have improved noticeably within recent years, among others due to developments in imaging techniques, endoscopic or computer-assisted surgery and intensity-modulated radiotherapy (IMRT) [1]. Nonetheless, a tradeoff between optimal tumor control and function preservation of organs at risk, e.g. the optic system, is frequently inevitable due to orbital or cranial nerve involvement. The biophysical properties of carbon ion RT (CIRT) enable both improved normal tissue sparing and dose escalation [6]. In addition, the increased relative biological effectiveness (RBE) and decreased oxygen dependence of CIRT are beneficial in radioresistant tumors [7,8]. Previous studies on CIRT of sinonasal cancer reported favorable local control and toxicity rates, compared to conventional RT for various tumor entities [[9], [10], [11]]. In a more recent publication from our center, the first prospective data on the toxicity and clinical outcomes of bimodal radiation therapy (CIRT + IMRT) for sinonasal malignancies were presented, specifically adenocarcinoma and squamous cell carcinoma, demonstrating a low incidence of acute toxicity alongside encouraging tumor control and survival rates [12]. However, clinical data on the effectiveness of CIRT for sinonasal SCC is scarce. In this context, carbon ion therapy offers distinct therapeutic advantages. Owing to their physical characteristics, carbon ions enable highly conformal dose distributions with sharp dose fall-off, which is particularly relevant in the anatomically complex paranasal sinus region where critical structures lie in close proximity. Beyond these physical benefits, carbon ions also exhibit increased relative biological effectiveness due to their higher linear energy transfer, resulting in increased cytotoxic efficacy even in more resistant histologies [[13], [14], [15]]. Also the management of the neck remains controversial in patients with sinonasal SCC, in particular for the node-negative-neck there is no established treatment standard [16]. The risk of nodal metastases in sinonasal SCC, primarily level Ib, IIa and VIIa, was reported to be around 7–22 % depending primarily on the tumor localization and T-stage [17]. Elective neck dissection can improve disease-specific survival in advanced stage maxillary sinus SCC [18] and is commonly recommended for T3–4 sinonasal cancer. Elective neck irradiation may reduce the rate of nodal recurrence in the cN0 situation [19] in advanced stage tumors. However, the question if a less radical approach with regard to the neck could be feasible to reduce toxicity in selected patients with advanced sinonasal SCC remains unclear. The aim of the current study was to investigate the treatment outcome, management of the neck and toxicity rates of IMRT with carbon ion boost in patients with sinonasal SCC.

## Methods

### Patient selection

After approval by the regional ethics committee (reference number S-421/2015, date of approval 18.06.2024), all patients with head and neck cancer treated by photon intensity-modulated radiotherapy (IMRT) with carbon ion boost at our institution were screened. All patients with squamous cell carcinoma (SCC) of the nasal cavity or paranasal sinuses treated between 2011 and 2019 were included. After exclusion of patients with incomplete clinical data or follow-up, a total of 43 patients were evaluated in the current study. A subgroup of 17 patients were treated within the IMRT-HIT-SNT phase II trial [12].

### Treatment planning

All patients were staged with CT of the neck and thorax and abdominal ultrasound. For precise delineation of the primary tumor and evaluation of the neck, a contrast-enhanced MRI of the neck was also performed.

Patients were immobilized in treatment position using thermoplastic head mask systems. The target volume delineation was conducted based on contrast-enhanced CT and MRI. The recommendations of the International Commission on Radiation Units and Measurements (ICRU) [20,21] were considered. Dose constraints of normal tissues were respected according to the Quantitative Analyses of Normal Tissue Effects in the Clinic [22].

The CTV boost included macroscopic tumor and/or the tumor bed with safety margins along typical pathways of spread. An isotropic margin of 3 mm was added for the PTV boost. The local effect model (LEM) I was applied for radiobiological modelling of the RBE of ions and calculation of the RBE-weighted dose [23,24]. An α/β value of 2 Gy was used for dose calculation with regard to the target and OAR [25,26]. The cumulative dose was specified by the equivalent dose in 2 Gy fractions (EQD2). The beam setup was individualized for each patient, optimized to mitigate range uncertainties caused by anatomical changes in the beam directions (e.g. mucosal swelling). CIRT was performed using active raster scanning technique. Daily orthogonal X-rays were used for image-guidance prior to irradiation followed by position correction. CIRT was applied with a single dose of 3 Gy (RBE) and 5–6 fractions per week. Most patients received the carbon ion boost at the beginning.

**Fig. 1:**
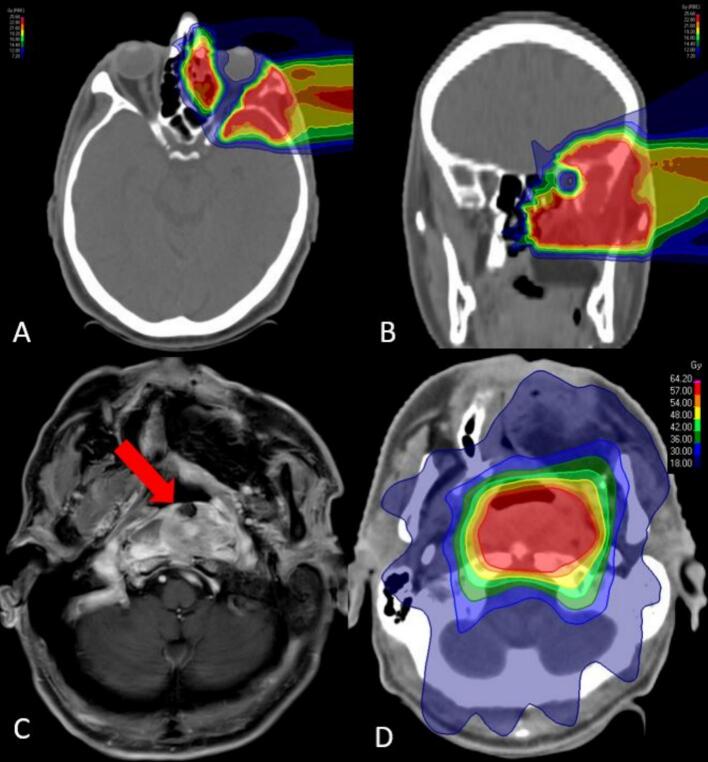
63-year-old male patient with a high-grade T4N0M0 squamous cell carcinoma of the paranasal sinuses. The patient received radiotherapy (RT) with 24 Gy (RBE) carbon ions (A + B) allowing for excellent preservation of the visual system followed by 50 Gy intensity-modulated RT (IMRT) after R0-resection. A local recurrence outside the clinical target volume of the carbon ion boost was diagnosed 21 months after RT (C). The patient received re-irradiation with a total dose of 60 Gy in 30 fractions IMRT (D).

The CTV included the CTV boost and involved anatomical sites as well as the locoregional lymph nodes. A margin of 3 mm was added for the PTV. Further dose levels were added in the case of diagnosed lymph node metastases. Image-guidance for IMRT consisted of daily MV cone-beam CTs. If required, the treatment plan was adapted to anatomical and positional changes. RT was delivered with a 6 MV-linear accelerator using the step-and-shoot IMRT, Volumetric Intensity Modulated Arc Therapy (VMAT) or Tomotherapy® technique. RT was applied with a single dose of 2 Gy and 5 fractions per week. A patient case is shown in Fig. 1.

### Follow-up

Clinical follow-up was conducted with MRI or CT of the head and neck 6–8 weeks after RT, every 3 months within the first 2 years, every 6 months in the third year and thereafter every year post-treatment. CT thorax and abdominal sonography was performed once per year. Toxicity was recorded non-standardized by a radiation oncologist according to CTCAE v5.0 and analyzed retrospectively. Patients presented either to the ear, nose and throat specialist or the maxillofacial surgeon for clinical examination in regular intervals.

### Statistical analysis

Statistical analysis was performed using R version 4.1.0 (https://www.r-project.org). Toxicity analysis was conducted using CTCAE v5.0. The mean number of toxicity events per patient was assessed based on the TAME method [27]. The mean number of grade III acute toxicity events per patient was compared between patients who underwent elective lymph node irradiation and those who did not, applying the Mann–Whitney *U* test. The median follow-up with regard to OS was determined using the inverse Kaplan-Meier method. OS, progression-free survival (PFS), local progression-free survival (L-PFS) and metastasis-free survival (MFS) were analyzed from the end of RT until death or tumor progression using the Kaplan-Meier method. Prognostic factors for OS were explored using the log-rank test. Multivariate analysis was inconclusive due to only few events during follow-up and therefore not evaluated.

## Results

### Patient and treatment characteristics

The median age before RT was 64 years (range 19–83 years) and most patients were male (n = 28; 65.1 %). The median time of hospitalization during RT was 7 days (range 0–53 days). The median time from initial diagnosis to RT was 3.1 months (range 1.0–93.1 months). A percutaneous gastric feeding tube was required in one patient (2.3 %). A total of 4 patients (9.3 %) had previous or synchronous and 2 patients (4.7 %) metachronous secondary malignancies, primarily in the aerodigestive tract. The majority of patients had G2 (n = 17; 39.5 %) or G3 (n = 24; 55.8 %) tumors. Distant metastases were present in 2 patients (4.7 %) prior to RT. Further patient and treatment characteristics are shown in Table 1.

**Table 1:** Patient and radiation treatment characteristics (n = 43 patients).

	**Patients**	**%**
**Sex**		
Female	15	34.9
Male	28	65.1
**Primary tumor localization**		
Paranasal sinuses	32	74.4
Nasal cavity	11	25.6
**T-stage prior to RT**		
T1-2	7	16.3
T3	7	16.3
T4	29	67.4
**Smoking**		
≥10 pack years	7	16.3
<10 pack years	1	2.3
Never	35	81.4
**RT setting**		
Additive	20	46.5
Definitive	18	41.9
Adjuvant	5	11.6
**Chemotherapy**		
Neoadjuvant	2	4.6
Simultaneous CRT	18	41.9
None	23	53.5
**Resection status**		
R0	8	25.8
R1	6	19.4
R2	7	22.6
RX	10	32.2
**Optimization strategy C12**		
Single beam optimization	3	7.0
Intensity-modulated particle therapy	40	93.0
**C12 gantry**		
yes	12	27.9
no	31	72.1
	**Median**	**Range**
Age prior to RT [years]	64	19–83
KPS prior to RT [%]	90	70–100
BMI prior to RT [kg/m^2^]	26.4	14.9–52.0
BMI post RT [kg/m^2^]	25.8	15.2–51.9
Hospitalization during RT [days]	7	0–53
Total dose IMRT [Gy]	50.0	50.0–60.0
Total dose C12 boost [Gy RBE]	24.0	12.0–24.0
Cumulative total dose [Gy EQD2]	80.0	75.0–80.0
RT fractions	33	32–34
Treatment time / fraction IMRT [min]	5.1	2.7–8.2
Treatment time / fraction C12 [min]	8.0	4.0–17.0
PTV [ccm]	312.5	111.5–845.9
PTV C12 boost [cm^3^]	166.0	41.2–503.0
CTV [cm^3^]	320.0	73.1–793.4
CTV C12 boost [cm^3^]	113.7	22.1–408.2
GTV [cm^3^]	49.5	4.2–214.0
Beam number	2	1–4
Total number of C12 scan spots	17,689	4,956–47,999
Maximum energy C12 [MeV/u]	247.7	182.0–297.8

Abbreviations: Radiotherapy (RT), chemoradiotherapy (CRT), Karnofsky Performance Score (KPS), body mass index (BMI), intensity-modulated RT (IMRT), relative biological effectiveness (RBE), equivalent dose in 2 Gy fractions (EQD2), planning target volume (PTV), clinical target volume (CTV), gross tumor volume (GTV).

**Fig. 2:**
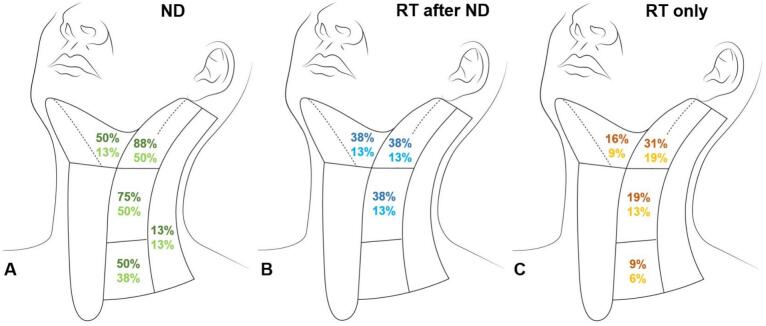
A total of 40 patients (93 %) had a clinical node-negative neck. Shown are the rates of ipsilateral (dark color) and bilateral (light color) elective neck treatment for each lymph node level in patients with neck dissection (ND; n = 8; A) ± radiotherapy (RT) after ND (B) and without ND (n = 32; C). None of the patients with elective ND had lymph node metastases. A total of 12 patients (30 %) received no elective neck treatment. The extent of elective treatment was lower in the RT only group compared to both ND only and RT after ND. In all patients treated with RT, the retropharyngeal lymph nodes where included.

The most common treatment regimen was 24 Gy (RBE) carbon ion boost in 8 fractions followed by 50 Gy IMRT in 25 fractions, consistent with a cumulative dose of 80 Gy EQD2. Most patients received postoperative (n = 25; 58.1 %) compared to definitive RT (n = 18; 41.9 %). A total of 31 patients (72.1 %) received surgical treatment at some point. The median number of surgical treatments prior to RT was 1 (range 0–4). Most patients (n = 40; 93.0 %) had no clinical evidence of lymph node metastases prior to treatment. In this cohort, 8 patients (20.0 %) received a neck dissection, none had detected lymph node metastases. Only one patient with tumor infiltration of the nasopharynx had diagnosed lymph node metastases in level II–III bilaterally. There were 22 different treatment constellations for the cervical lymph nodes, which are summarized in Table 2/Fig. 2.

**Table 2:** Clinical management of the cervical lymph nodes in patients with sinonasal SCC.

	**Pat.**	**cN + levels**	**ND levels**	**pN + levels**	**RT levels**	
**Node negative neck (n = 40)**						
Ipsilateral ND (n = 4)	1	None	I-III	none	I-III + RP	ipsilat.
	1		I	none	none	n.a.
	1		I-II	none	none	n.a.
	1		II-IV	none	none	n.a.
Bilateral ND (n = 4)	1		II-V	none	I-III + RP	bilat.
	1		II-IV	none	I-III + RP	ipsilat.
	1		I-III	none	none	n.a.
	1		II-IV	none	none	n.a.
No ND (n = 32)	12		none	n.a.	none	n.a.
	4		none	n.a.	I-IV + RP	bilat.
	4		none	n.a.	II + RP	bilat.
	1		none	n.a.	I-II + RP	bilat.
	1		none	n.a.	II-III + RP	bilat.
	1		none	n.a.	I-III + RP	bilat.
	1		none	n.a.	II-IV + RP	bilat.
	4		none	n.a.	II-III + RP	ipsilat.
	2		none	n.a.	I-IV + RP	ipsilat.
	1		none	n.a.	I-II + RP	ipsilat.
	1		none	n.a.	II + RP	ipsilat.
**Node positive neck (n = 3)**						
Ipsilateral neck dissection	1	II ipsilat.	I-III ipsilat.	none	I-III + RP	ipsilat.
Bilateral neck dissection	1	II-IV ipsilat.	II-IV	none	I-IV + RP	ipsilat.
No neck dissection	1	II-III bilat.	none	n.a.	II-IV + RP	bilat.

Abbreviations: Squamous cell carcinoma (SCC), neck dissection (ND), radiotherapy (RT), retropharyngeal (RP).

### Treatment outcome

The median follow-up with regard to OS after RT was 25.1 months (range 2.0–100.0 months).

**Fig. 3:**
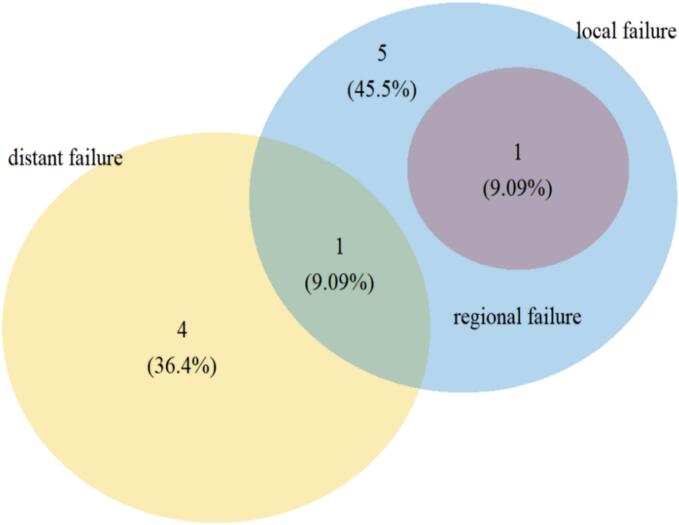
Venn diagram showing the pattern of local, regional and distant failure for all patients with progressive disease after radiotherapy (n = 11; 25.6 %), analyzed regarding the site of first failure only. Most patients developed local failure (n = 7; 63.6 %), the majority within the clinical target volume of the carbon ion boost (n = 4; 36.4 %). Only one patient developed regional failure.

The one- and two-year PFS was 79.1 % and 75.5 %, respectively (supplementary figure 1). Most patients with disease progression after RT developed local failure (n = 7; 63.6 %) and 4 of these local relapses (36.4 %) occurred within the carbon ion boost CTV. Regional failure occurred in one patient who also developed simultaneous local failure. The pattern of failure is shown in Fig. 3.

The OS, L-PFS and MFS after 12/24 months were 93.0 %/89.3 %, 88.4 %/79.0 %, and 86.0 %/86.0 % (supplementary figure 2–4). The total hospitalization days were the only prognostic factor associated with decreased OS (p = 0.012; supplementary figure 5). Further factors, among others CRT vs. RT, postoperative vs. definitive RT or the Karnofsky Performance Score had no significant impact on OS.

### Acute and late toxicity

Radiotherapy was discontinued in one patient (2.3 %) due to newly diagnosed brain metastases during treatment. All other patients could complete RT without interruptions. The cumulative rate of acute grade III toxicity was 9.3 % (n = 4). Acute grade III toxicity events included oral mucositis, radiation dermatitis and weight loss. The mean number of acute grade I and II toxicity events per patient was 1.7 (95 %-confidence interval [CI] 1.2–2.1) and 3.1 (95 %-CI 2.5–3.8). The most common grade I–II acute toxicities were oral mucositis (n = 28; 65.1 %), dysphagia (n = 25; 58.1 %) and radiation dermatitis (n = 22; 51.2 %). The mean number of grade III acute toxicity events per patient was higher if elective lymph node irradiation was performed (0.24 vs. 0.03; p = 0.181).

The cumulative rate of late grade III toxicity was 4.7 % (n = 2). Late grade III toxicities included dysphagia and hearing impairment. The mean number of late grade I and II toxicity was 1.7 (95 %-CI 1.3–2.1) and 0.7 (95 %-CI 0.4–0.9). The most common grade I–II late toxicities were dry mouth (n = 18; 41.9 %), dysgeusia (n = 16; 37.2 %) and lymph edema (n = 13; 30.2 %). No grade IV acute or late toxicity was reported. Further details on the mean number and total counts of acute and late toxicity events are summarized in Table 3a, Table 3b.

**Table 3a:** Mean number of CTCAE v5.0 toxicity events and overall toxicity per patient (n = 43).

	**Mean**	**95 %-CI**	**Patients**	**%**
**Acute short-term toxicity**				
CTC grade I	1.7	1.2–2.1	1	2.3
CTC grade II	3.1	2.5–3.8	38	88.4
CTC grade III	0.1	0–0.2	4	9.3
				
**Late toxicity**				
CTC grade I	1.7	1.3–2.1	16	37.2
CTC grade II	0.7	0.4–0.9	17	39.5
CTC grade III	0.1	0–0.1	2	4.7

Abbreviations: Common Terminology Criteria of Adverse Events (CTCAE), confidence interval (CI); Comment: If a patient had more than one toxicity event, only the highest toxicity grade was counted once in the total count category.

**Table 3b:** Most common CTCAE v5.0 grade I-III acute short-term and late toxicities (n = 43).

	**Acute toxicity**			**Late toxicity**		
	**° I**	**° II**	**° III**	**° I**	**° II**	**° III**
Oral mucositis	4 (9.3 %)	24 (55.8 %)	3 (7.0 %)	3 (7.0 %)	0	0
Dysphagia	7 (16.3 %)	18 (41.9 %)	0	4 (9.3 %)	1 (2.3 %)	1 (2.3 %)
Radiation dermatitis	11 (25.6 %)	11 (25.6 %)	1 (2.3 %)	4 (9.3 %)	0	0
Dry mouth	5 (11.6 %)	4 (9.3 %)	0	16 (37.2 %)	2 (4.7 %)	0
Dysgeusia	1 (2.3 %)	10 (23.3 %)	0	13 (30.2 %)	3 (7.0 %)	0
Fatigue	6 (14.0 %)	6 (14.0 %)	0	9 (20.9 %)	0	0
Mucosal infection	0	10 (23.3 %)	0	0	0	0
Lymph edema	2 (4.7 %)	3 (7.0 %)	0	7 (16.3 %)	6 (14.0 %)	0
Weight loss	5 (11.6 %)	6 (14.0 %)	1 (2.3 %)	1 (2.3 %)	4 (9.3 %)	0
Dry eye	2 (4.7 %)	7 (16.3 %)	0	0	0	0
Nausea	2 (4.7 %)	7 (16.3 %)	0	0	0	0
Dehydration	2 (4.7 %)	7 (16.3 %)	0	0	0	0
Hearing impariment	3 (7.0 %)	3 (7.0 %)	0	4 (9.3 %)	3 (7.0 %)	1 (2.3 %)
Conjunctivitis	3 (7.0 %)	4 (9.3 %)	0	1 (2.3 %)	2 (4.7 %)	0
Trismus	2 (4.7 %)	2 (4.7 %)	0	4 (9.3 %)	1 (2.3 %)	0
Optic nerve disorder	1 (2.3 %)	0	0	2 (4.7 %)	2 (4.7 %)	0

Abbreviations: Common Terminology Criteria of Adverse Events (CTCAE), Comment: Acute toxicity that continued in the late phase was counted as both acute and late toxicity.

## Discussion

The current study reports first clinical results of IMRT with carbon ion boost in patients with high-risk sinonasal SCC. Radiotherapy yielded promising early tumor control rates and was associated with favorable toxicity. Treatment decisions with regard to the management of node-negative-neck were challenging and highly individualized.

The reported results raise the question whether dose escalation using IMRT with carbon ion boost could improve local control in high-risk sinonasal SCC. Our findings indicate better local control compared to historical cohorts, where local recurrence rates after surgery and postoperative RT approached 50 % at five years [28]. The only prospective study on CIRT for sinonasal cancers reported 2-year OS and L-PFS rates of 79.4 % and 61.8 %, respectively [12]. Our study seems to be in line with these results, showing 2-year OS and L-PFS of 89.3 % and 79.0 % after a carbon ion boost, suggesting that this strategy leads to favorable outcomes compared to historical cohorts.

A major challenge in the treatment of sinonasal squamous cell carcinoma (SCC) remains the high rate of local failure, which persists even with the integration of a carbon ion boost. In our series, more than one-third of local recurrences occurred within the carbon ion boost volume, underscoring that while CIRT achieves excellent dose conformity and biological effectiveness, there is still considerable room for improvement in local control. By contrast, nodal failures were infrequent, regardless of the heterogeneous management strategies applied, suggesting that the primary barrier to long-term disease control in this setting continues to lie within the local tumor compartment.

It is important to recognize that CIRT is a relatively young technique and remains under continuous technical and biological refinement. One potential strategy for further optimization is the underlying radiobiological modeling of the relative biological effectiveness (RBE). In our cohort, dose prescription was based on the Local Effect Model I (LEM I). However, other models are in clinical use, including the modified microdosimetric kinetic model (mMKM) and the MKM-derived Japanese biological model (NIRS-MKM), each of which results in different RBE-weighted dose distributions. Recent developments in treatment planning allow for mixed- or multi-RBE optimization, where distinct RBE models can be applied to different regions of interest [29]. Such strategies may enable a more accurate biological dose calculation, particularly in the context of heterogeneous tumor volumes or advanced-stage disease, where dose–effect relationships may differ substantially. These ongoing refinements highlight that current clinical outcomes with CIRT should be interpreted within the framework of evolving radiobiological models. In particular, given that the majority of patients in our cohort presented with locally advanced SCC, further research is warranted to determine whether such subgroups − especially those with larger tumor volumes or higher-stage disease − derive a specific benefit from this approach. Whether the proposed carbon ion boost regimen is the most appropriate approach to improve local control, compared with definitive hypofractionated CIRT, cannot be determined from this study, as only indirect comparisons with previously published data are possible. Prospective randomized studies are needed to clarify this question and to establish evidence-based treatment standards.

In addition, our results provide further clinical evidence that biophysical benefits of CIRT might translate into reduced toxicity rates compared to conventional RT in patients with sinonasal cancer. Despite precision advantages of IMRT [30] and complication reduction by endoscopic surgery [31], a benefit-risk-tradeoff between tumor control and organ function preservation is often required. In particular elderly patients are at risk for increased toxicity [32]. Historic series provide important context for toxicity outcomes. Suh et al. reported a markedly lower incidence of acute grade 3 mucositis with IMRT (16 %) compared to 3D-RT (37 %) [33]. In a pooled analysis of available studies, Zhang et al. observed slightly higher rates of acute grade 3–4 toxicity following CIRT (>30 %) relative to IMRT (24 %) [9]. Across treatment modalities, late toxicity was consistently reported in the range of 10–13 %. On the contrary, favorable toxicity rates of IMRT with carbon ion boost were previously reported for various sinonasal tumors, among others adenoid cystic carcinoma, adenocarcinoma and malignant melanoma [10,34,35]. The rate of grade III toxicity ranged between 0–40 %. In line with these studies, the rate of acute and late grade III toxicity was 9.3 % and 4.7 % in the current analysis. Considering the high-risk tumor characteristics, the mean number of grade I–II acute and late toxicity events per patient of 4.8 and 2.4 was acceptable. Nevertheless, the ability to ascertain whether the addition of a carbon ion boost confers a clinically meaningful reduction in toxicity compared with IMRT remains limited, owing to the retrospective design and the non-standardized nature of toxicity assessment in our study. Although non-significant, there was a trend towards fewer grade III toxicity events in patients without RT of the lymph nodes. Evaluated in prospective clinical trials, improved toxicity rates through reduced volumes of lymph node RT were previously reported [36]. However, any approach to reduce toxicity based on a less intense treatment of the neck should consider the potentially increased risk for regional recurrence. In the case of positive lymph nodes, there is a significant adverse effect on local control, distant spread and OS [17,37].

The management of the neck was highly individualized in the current study with a total of 22 different treatment constellations. Although patients could benefit from a personalized approach in general, evidence-based treatment algorithms are required. Most patients in our study had cN0 status (n = 40; 93.0 %) and only one patient (2.3 %) with tumor extension to the nasopharynx initially had diagnosed lymph node metastases. In patients with maxillary sinus SCC and initial cN0 status (81–94 % % of the patients), the rate of regional recurrence was reported between 3–30 % without elective lymph node irradiation [37]. The regional control was excellent in the current study since only one patient developed combined local and nodal failure. However, most patients (n = 30; 69.8 %) received elective neck treatment, 25 patients (58.1 %) either neck dissection or RT and 5 patients (n = 11.6 %) both. Elective neck treatment with RT was generally less extensive than with ND ± RT. Although prophylactic treatment of the node-negative-neck remains controversial [28], it is generally recommended in locally advanced sinonasal SCC (T3–4 stage) due to the risk of nodal involvement [18]. Tumor localization in the maxillary sinus and extension to the oral cavity or nasopharynx are further risk factors for nodal spread [17]. For selected patients with N0 status and low risk for nodal involvement, deescalation of the lymph node treatment could be beneficial. The most obvious possible measure is to treat advanced stage node-negative sinonasal SCC with either elective neck dissection or nodal irradiation but not both. The reported rates or regional recurrence after neck dissection without additional lymph node RT were reported around 5–15 % [38,39]. Selective neck dissection may be feasible to detect occult lymph node metastases in sinonasal SCC [40], but does not improve neck control [38]. The rate of neck recurrence after elective nodal irradiation without prior neck dissection was reported around 0–5 % [19,41,42]. Further possible deescalation strategies include the reduction of irradiated lymph node volume based on a certain threshold of nodal involvement for each level [43]. Besides rarely involved lymph node levels (e.g. IV–V) in node-negative tumors, irradiation of the contralateral neck can be omitted in well-lateralized tumors without contralateral neck involvement. However, data to support this treatment algorithm in sinonasal SCC is scarce, based on retrospective studies and inhomogeneous, e.g. with regard to diagnostic workup. Furthermore, physiological lymph node drainage routes are often individual and complex and can be influenced among others by surgery or tumor extension [44]. Therefore, risk-adapted neck irradiation guided by lymph node scintigraphy could enhance consistency, e.g. with regard to the controversial levels VIIa and IX. Also, photoacoustic molecular imaging shows potential to detect occult metastatic lymph nodes in patients with head and neck cancer [45]. Further clinical evidence using modern imaging, radiation techniques and patient reported outcomes is required to improve evidence-based treatment algorithms of the node-negative-neck in sinonasal SCC.

### Limitations

The current study had several limitations. First, the toxicity was recorded non-standardized for most of the patients. Second, further long-term follow-up is required to evaluate treatment outcome. Third, the treatment regimens with regard to the management of the neck were rather heterogeneous. Despite these limitations, the current study reported encouraging results, thereby adding valuable evidence to the recent developments in the radiation treatment of high-risk sinonasal SCC.

## Conclusions

In the current study, first clinical results of IMRT with carbon ion boost in patients with high-risk sinonasal SCC showed encouraging early local tumor control and OS. The management of the node-negative-neck was highly individualized. Toxicity rates were encouraging and could be further reduced by a less radical treatment approach of the cervical lymph nodes in selected patients.

## Funding support

T.H., M.D. and F.S. are funded by the Physician-Scientist Program of Heidelberg University, Faculty of Medicine.

## CRediT authorship contribution statement

**Lukas Bauer:** Data curation, Investigation, Validation, Methodology, Visualization, Writing – original draft, Writing – review & editing. **Katharina Weusthof:** Data curation, Investigation, Validation, Methodology, Writing – review & editing. **Sebastian Regnery:** Data curation, Investigation, Validation, Methodology, Writing – review & editing. **Maximilian Deng:** Data curation, Investigation, Validation, Methodology, Writing – review & editing. **Philipp Schröter:** Data curation, Investigation, Validation, Methodology, Writing – review & editing. **Florian Stritzke:** Data curation, Investigation, Validation, Methodology, Writing – review & editing. **Nils Netzer:** Data curation, Investigation, Validation, Methodology, Writing – review & editing. **Henrik Franke:** Data curation, Investigation, Validation, Methodology, Writing – review & editing. **Kristin Uzun-Lang:** Data curation, Investigation, Validation, Methodology, Project administration, Supervision, Writing – original draft, Writing – review & editing. **Julius Moratin:** Data curation, Investigation, Validation, Methodology, Project administration, Supervision, Writing – original draft, Writing – review & editing. **Oliver Ristow:** Data curation, Investigation, Validation, Methodology, Project administration, Supervision, Writing – original draft, Writing – review & editing. **Rubens Thoelken:** Data curation, Investigation, Validation, Methodology, Project administration, Supervision, Writing – original draft, Writing – review & editing. **Semi B. Harrabi:** Data curation, Investigation, Validation, Methodology, Project administration, Supervision, Writing – original draft, Writing – review & editing. **Klaus Herfarth:** Data curation, Investigation, Validation, Methodology, Project administration, Supervision, Writing – original draft, Writing – review & editing. **Sebastian Adeberg:** Data curation, Investigation, Validation, Methodology, Project administration, Supervision, Writing – original draft, Writing – review & editing. **Jürgen Debus:** Data curation, Investigation, Validation, Methodology, Project administration, Supervision, Writing – original draft, Writing – review & editing. **Thomas Held:** Data curation, Investigation, Validation, Methodology, Visualization, Writing – original draft, Project administration, Supervision.

## Declaration of competing interest

The authors declare the following financial interests/personal relationships which may be considered as potential competing interests: J.D. reports grants from CRI The Clinical Research Institute, grants from View Ray Inc., grants from Accuray International, grants from Accuray Incorporated, grants from RaySearch Laboratories AB, grants from Vision RT limited, grants from Merck Serono GmbH, grants from Astellas Pharma GmbH, grants from Astra Zeneca GmbH, grants from Siemens Healthcare GmbH, grants from Solution Akademie GmbH, grants from Eromed PLC Surrey Research Park, grants from Quintiles GmbH, grants from Pharmaceutical Research Associates GmbH, grants from Boehringer Ingelheim Pharma GmbH Co, grants from PTW-Frieburg Dr. Pychlau GmbH, grants from Nanobiotix A.a., outside the submitted work. The other authors declare no conflict of interest. S.A. reports grants from Novocure Inc., Accuray International Sàrl, Merck Sharp&Dohme, Sanofi-Aventis GmbH and Merk Serono GmbH outside the submitted work.
