# Supplementary material for: Intensity-modulated radiotherapy with carbon ion boost for high-risk sinonasal squamous cell carcinoma: clinical outcomes and the management of the node-negative neck
Source: Clin Transl Radiat Oncol. 2025 Oct 27;56:101064. doi: 10.1016/j.ctro.2025.101064 (PMC12639251; doi:10.1016/j.ctro.2025.101064)
Supplement: Supplementary Data 1 [file mmc1.docx]

Supplementary Materials

Figure 1: Progression-Free Survival (PFS)

Figure 2: Overall Survival

Figure 3: Local Progression-Free Survival (LPFS)

Figure 4: Metastasis-Free Survival (MFS)


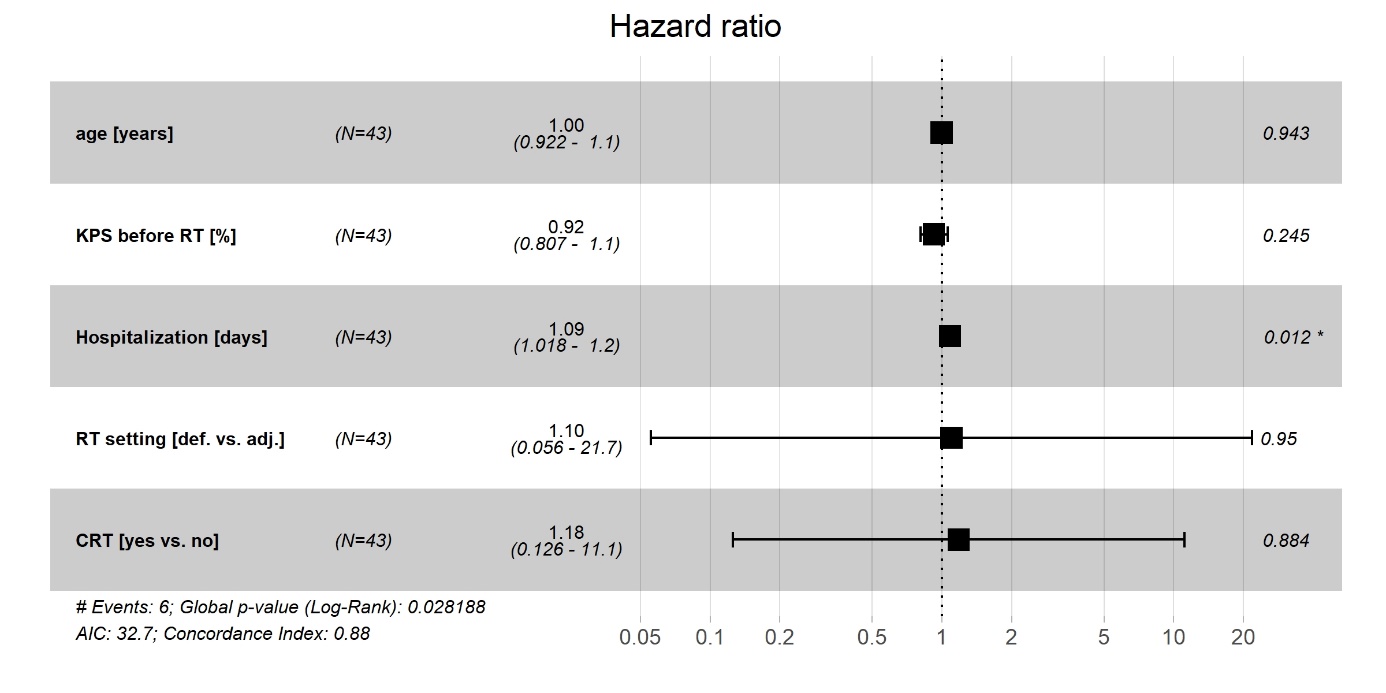


Figure 5: Prognostic factors for Overall Survival: Log-Rank Test. KPS: Karnofsky Performance Score; RT: Radiotherapy; def.: definitive; adj.: adjuvant; CRT: Chemoradiotherapy
